# A Model of Perceived Co-creation Value in Tourism Service Setting: An Application of Structure Equation Modeling

**DOI:** 10.3389/fpsyg.2022.808114

**Published:** 2022-02-25

**Authors:** Kefang Tao, Jiangeng Ye, Hanjie Xiao, Poju Chen

**Affiliations:** ^1^School of Tourism, Kunming University, Kunming, China; ^2^Faculty of Humanities and Social Science, Yunnan Agriculture University, Kunming, China; ^3^Business School, Huzhou University, Huzhou, China; ^4^School of Business, North Carolina Central University, Fayetteville, NC, United States

**Keywords:** perceived value, value co-creation, scale development, semi-structured interview, structure equation model, customer satisfaction, customer loyalty

## Abstract

This study explores how the perceived co-creation values (PCVs) from tourists’ perspectives are applied in the customized tour arrangement service setting. The sequential qualitative and quantitative methods are adopted for this study. The initial qualitative method in terms of the proactive semi-structured interview is conducted to identify and explore the dimension of the PCV construct and to develop its measurement scale. The quantitative method by the structure equation model is employed for the proposed conceptual model fitness assessment and consolidation. Our work contributes to the progression of value co-creation research in a customized tourism context and provides a valid and reliable PCV instrument to tourism practitioners for a better service platform designing. The mediating role of customer satisfaction (CS) between PCV and customer loyalty (CL) offers service providers a deeper understanding of customer psychology and behavior, and thus, the loyal customer cultivation strategy.

## Introduction

As we step into the era of the experience economy, increasing attention has been received, especially by a high-contact service company. Although experience is intangible and immaterial, it is more impressive and can “touch” people better than products or services. People attached great value to it because they are memorable (Andersson, 2007) and more likely to be unforgettable even for the lifetime. Tourism is one of the biggest experience generators and one of the greatest and ever growing sources of experiences with which people construct their own unique narratives, consequently, this line of thought deserves our attention.

In the tourism context, it is of paramount importance for tourism service suppliers to remain competitive by providing customers with a unique and memorable experience, which requires customer participation and a connection that links the customer to the experience (Pine and Gilmore, 1998; Shaw et al., 2011). Tourism scholars believe that the concept of co-creation is particularly relevant. The most frequently quoted definition of value co-creation is proposed by Prahalad and Ramaswamy (2004), who view value co-creation as an aggregation of customers and as an exchange of service and product offerings. Traditional economics places value extraction at the key point of the interaction by firm and customer, while in the co-creation perspective, the whole process of interaction is opportunities for both value extraction and creation.

Customization, which has focused on customers’ individual needs, has been considered as one of the latest field of marketing by Professor Philip Kotler in the twenty-first century (Kotler and Keller, 2006). A part of consumers who are informed, networked, empowered, and active, are no longer satisfied with the passive acceptance of the tourism product or service. Young independent travelers are willing to construct their travel plans, which offer choices for transportation, accommodations, attractions, meals, shopping, entertainment, etc. The future will belong to those travel professionals who can successfully coordinate and co-create with customers and deliver them with optimal travel products and memorable experiences. Thus, the co-creation of customized package tours benefits one-to-one marketing for personalized and differentiated products and services in the tourism industry.

Limited empirical research has been done to investigate the dynamic experience in affecting the phenomenon of co-creation values between service providers and tourists. Our study focuses on customized tour arrangements as an appropriate setting to illustrate the value co-creation of the touristic experience.

Differentiated from previous studies that frequently and solely investigate the driving forces of value co-creation by customer participation (Chan et al., 2010; Dabholkar, 2015; Assiouras et al., 2019) and company support (Prahalad and Ramaswamy, 2004; Grissemann and Stokburger-Sauer, 2012; O’Cass and Sok, 2015), our research takes an insight into the longitudinal investigation of value co-creation dimensions and its outcomes from the customer perspective in customized tour operation, which is expected to extend the understanding of value co-creation’s dimensionality and its effect on tourists’ perception. Our work articulates co-created value formation by examining influencing factors and the consequent variable of consumer satisfaction and loyalty. Latent variables are integrated into a proposed framework, and the dynamic relationships between each of them are measured statistically.

Additionally, our research measured tourists’ actual degree of value co-creation by distributing the questionnaires to customers once they had booked their travel package. This operation distinguishes the present study from previous ones that measured the customer’s willingness to engage in co-creation (Ophof, 2013; Yen, 2015), or that there will be a time lag that participants’ responses might be influenced by their pre-existing travel experience (Tu and Zhang, 2013). Rather et al. (2022) conducted a first-time vs. repeat tourism customer engagement, experience, and value co-creation study. Their investigation concentrates on customer tourism destination-related experience, while our work focuses on customized tour arrangement service setting.

Furthermore, the vast majority of value co-creation studies have been conducted in the Western context. To the authors’ knowledge, China’s tourism industry has been largely neglected despite the booming e-Tourism industry in mainland China. This is another one of the motivator for conducting this study.

The present study is built on the foundation of resource exchange theory (Foa and Foa, 1974). It was developed to examine the patterns and types of exchange behavior involved in interpersonal interactions, which were performed by both sides of service provider and customers in this study. Similarly, Hare et al.’s (1959) social exchange theory is applied to better understand the driving forces behind the high contact interaction and the valuable outcomes consequently. Besides, social identity theory (Tajfel and Turner, 1979) explains why the specific groups of customers are willing to be involved in the co-creation activity launched by a service provider. The contributions adding to the above existing theories by applying them to co-creation activity in tourism in this study were discussed in detail in the theoretical application section.

## Research Process

### Research Design

This study attempts to offer some suggestions for customized tour marketing practitioners to increase customer positive psychological perception through value co-creation activity and further strategy of loyalty customer cultivation. In considering the research objectives and the complexity of the research, a rigorous research design is required.

As research methodology continues to evolve and develop, mixed-methods that employ the combination of qualitative and quantitative approaches are another step forward and gain its popularity. This popularity is because problem addressed by social and human sciences is complex, utilizing the strengths of combined qualitative and quantitative research provide and expand the problem understanding, also incorporate the need both to explore and explain (Creswell, 2009).

The mixed-methods of qualitative and quantitative approaches were adopted for current research. A sequential study with the qualitative method as phase one is for perceived co-creation values (PCVs) dimensions exploring and identifying and its measurement scale development. Phase two focused on statistical analysis, using the quantitative method for data examination, hypotheses testing, and the overall model consolidation.

### Qualitative Research

#### Semi-Structured Interview

**FIGURE 1:**
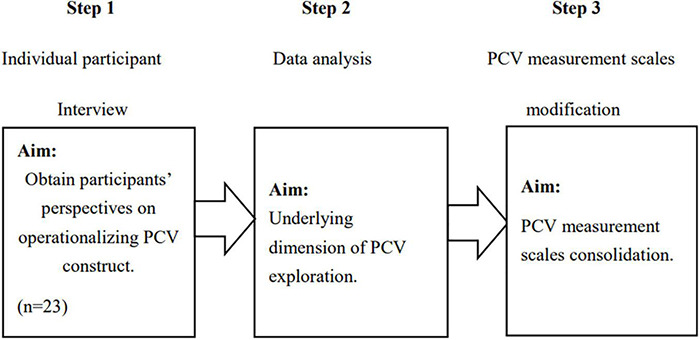
Procedures of qualitative research (*n* = 23). PCV, perceived co-creation values.

The exploratory qualitative methods are conducted for the potential dimension of PCV identification. Particularly, this study focused on semi-structured interviews for this step (Figure 1). The purpose of the interviews was to obtain participant perspectives on the PCVs construct as defined in the initial measurement list developed from the extant literature.

Semi-structured interviews were selected to realize the potential dimension based on two primary considerations. First, the interviews must be suitable for exploring respondent perceptions and opinions of complex issues, enabling the discovery of additional information relevant to the research topic. Second, compared to standardized interviews, semi-structured interviews allow respondents to express opinions more freely. This is because structured interviews use the same words repeatedly, which may overlook variances in respondent vocabulary levels, which may impact the validity and reliability of data obtained (Barriball and While, 1994).

#### Data Collection and Analysis

In the current study, researchers conducted semi-structured interviews among a small sample group consisting of seventeen experienced tourists and six seasoned tourism practitioners (see Table 1). Considering their seniority and professional experience, the informants were deemed appropriate and knowledgeable for the survey.

**TABLE 1:** Profile of interviewees for semi-interviews.

No.	Gender	Age	Occupation	Education level
01	Male	34	Student	Ph.D.
02	Female	37	Journalist	Postgraduate
03	Male	28	Bank clerk	Postgraduate
04	Female	31	IT sales	Undergraduate
05	Male	27	Civil servant	Undergraduate
06	Male	26	Self-employed	Undergraduate
07	Male	35	Designer	Undergraduate
08	Male	29	Nurse	Undergraduate
09	Male	27	Private business owner	Undergraduate
10	Male	35	Doctor	Postgraduate
11	Male	30	Teacher	Postgraduate
12	Female	25	Company administrator	Postgraduate
13	Male	35	Private business owner	Undergraduate
14	Female	34	Self-employed	Undergraduate
15	Female	34	NGO employee	Postgraduate
16	Female	34	Lecturer	Ph.D.
17	Female	34	Self-employed	Undergraduate
18	Male	45	Founder of travel agency	Undergraduate
19	Female	34	Travel agency sales	Undergraduate
20	Female	24	Tourism planner	Undergraduate
21	Female	32	Travel agency manager	Undergraduate
22	Female	38	Founder of travel agency	Undergraduate
23	Male	34	Travel agency sales director	Postgraduate

Rich and in-depth information were provided about the PCV of a customized tour. The interviews were administered in person by the researchers. The process of the interviews was audio recorded with the grants of the informants. Each of the interviews took approximately 40 min. To ensure the accuracy, the transcripts were cross-checked (Decrop, 1999) by the authors. The approach of thematic analysis was adopted for data analysis (Boyatzis, 1998). Themes were extracted from the data based on the information captured. The participants were well explained about the definition of value co-creation and its general practices in tourism service before the interview. To elicit the perspectives and knowledge of participants, two simple open-ended questions are prepared:

RQ1: To what extent do you agree with the listed items representing the construct of PCVs?

RQ2: Are there other values you would consider as important outcomes of co-creation activity?

The researchers analyzed and summarized suggestions and comments from the interviewees to revise the measurement scales for assessing the PCV construct. The details are delineated below.

The interviewees generally agreed with the initially proposed eight items representing PCVs. However, a portion of participants responded with additional experience and knowledge insights. The key informants reported as:

“*I will be able to make a deal with the sales agent more efficiently and effectively.*” (No. 1, male, 34, Ph.D. student).

The pattern of these interactions and a deep understanding of the content are necessarily included in organizational learning (Grönroos, 2006).

“*For me, it pushed me to explore my real travel expectations; the supplier gets a better understanding of its consumers, identifies trends, assesses consumer desires, and preferences more accurately.*” (No. 14, female, 34, self-employed).

Communications should seek to influence practices to assist customers to better utilize resources, not only the customers’ own resources but also those of the suppliers (Payne et al., 2008).

“*We know each well, I get familiar with their products, it helps me to better manage it. Moreover, on the one hand, the quotation sets my travel budget, on the other, the travel agency could make a better judgment of my consumption ability.*” (No. 15, female, 34, NGO employee).

“*I received experience. My knowledge will be richer and more comprehensive the next time I need to arrange my own travel or I am consulted by others.*” (No.2, female, 37, journalist).

Similar responses were expressed by tourism practitioners:

“*They more we communicate, the better we know each other. I will be able to serve my next customer better and more effectively, which benefits the customers.*” (No. 24, male, 34, travel agency sales director).

Co-creation can be regarded as a process of information and knowledge exchange, during which both the customer and firm learn from each other as the key components of co-creation (Payne et al., 2008). Customer learning behaviors can occur at different levels of process complexity. Remembering, internalization, and proportioning are the three levels of customer learning. Traditionally, remembering is emphasized by marketing communications. Compared with customers’ abilities to process emotions and information, remembering is about customers’ attention and is the initial form of learning. Internalization is the second level of customer learning. Customers interpret and assimilate information and messages during this process. The third form of customer learning is referred to as “proportioning,” which is more complex. Argyris and Schön (1978) believed that proportioning is “double-loop learning.” It allows the customer to take “one step backward” to review how they engage with suppliers. Because of such reflection, their behavior may change and differ from existing practices by performing new activities or disengaging and utilizing resources in new ways. That is, at the first level, the consumer’s attention is focused on attractive tourism information provided by the travel agency without processing (i.e., they are just simply remembering). At the second level, information is internalized by consumers, which results in a knowledge increasing experience. Finally, consumers correct chronic self-behaviors to utilize resources efficiently. Thus, customers benefit from the process of knowledge and information exchange in terms of value co-creation activity. Table 2 summarizes the findings from the semi-structured interviews.

**TABLE 2:** Revisions based on semi-structured interviews.

Variable of interest	Initial efforts	Revisions with inputs from participants
Perceived co-creation values	No item representing customer evaluation of the information provided by service supplier.	Additional item “The information and knowledge provided by travel agency is attractive.”
	No item relating to customer knowledge increase.	Additional item “It helps to increase my knowledge.”
	No item relating to customer experience increase.	Additional item “It helps to enrich my experience.”
	No item representing customer behavior changes	Additional item “It helps me amend some of my chronic behavior.”
		Additional item “It helps me utilize the resource efficiently.”

After the interviews, the researcher further modified the measuring scales of the PCV construct. Therefore, five additional items representing PCVs were added to the previously proposed eight items (Table 3).

**TABLE 3:** Interview summary.

No.	Measurement items stand for PCV	Number of informants who refer to the items
1	It helps me receive higher quality services.	23
2	It helps me receive more customized services.	23
3	It helps to make the products and services closer fit with my needs.	23
4	It helps me receive more control over the services quality.	11
5	It helps to reduce service failure.	14
6	It helps me build a better relationship with the service provider.	23
7	It helps to makes the service interaction more enjoyable.	14
8	It helps me receive relational approval from the service provider.	23
9	The information and knowledge provided by travel agency is attractive.	14
10	It helps to increase my knowledge.	14
11	It helps to enrich my experience.	16
12	It helps me amend some of my chronic behavior.	11
13	It helps me utilize the resource efficiently.	12

*PCV, perceived co-creation values.*

### Quantitative Research

The study was performed by using a purposive sampling method. The entity being analyzed is the individual customer of a specific customized tour. The questionnaire distribution was performed by researchers at two tour service companies for 3 months. Self-administrated surveys were conducted with 800 questionnaires, 732 useable questionnaires were collected with the total response rate at 91.5% (no missing data). In aims to measure customers’ actual degree of co-creation, respondents were invited to be involved in questionnaire filling once they finished their travel package booking. Thus, their responses would not be influenced either by their later travel experience or by the time lag between the co-creation activity and the survey.

The usable questionnaires were analyzed using the Statistical Package for Social Sciences software (SPSS 21). Corrected-Item Total Correlation (CITC) and Cronbach’s alpha were utilized to test the reliability of the items of a certain scale (Nunnally, 1994). Kaiser-Meyer-Olkin (KMO) and Bartlett’s Test of Sphericity were employed for the validity test (Churchill, 1979).

#### Hypotheses Development

##### Direct Relationships of Perceived Co-creation Values and Tourist Loyalty

As the highly competition environment in marketing, retaining, and cultivating customer loyalty (CL) has become increasingly vital. Numerous empirical research studies have been conducted for CL development.

Hospitality scholars demonstrate that customer co-creation value displays an enhanced loyalty intent in customers’ destination-related experience (Rather et al., 2022). More specifically, rather than take co-creation value as a first-order construct, we consider it as a second-order variable with three proposed dimensions and try to clarify the relationship of each of its dimension to CL, respectively. Hospitality firms could communicate value to customers by establishing the economic value (EV) and unique attributes of their products and services to win CL (Victorino et al., 2005). More relational benefits have been verified to have a direct effect on CL in retailing industry (Chen and Hu, 2010). Similarly, Chang (2013) declared that corporate reputation enhances CL through perceived trust and customer perceived value. Therefore, we assume that:

**H1.** Perceived co-creation of EV is positively and directly related to CL.

**H2.** Perceived co-creation of relational value (RV) is positively and directly related to CL.

**H3.** Perceived co-creation of information/knowledge value is positively and directly related to CL.

##### Effect of Perceived Co-creation Values on Tourist Satisfaction

Empirical studies that generally support the relationship between customization and satisfaction can be found in previous works. Regarding service experiences, Vega-Vazquez et al. (2013) interviewed 547 customers using personal care services, they identified that value co-creation behavior directly enhanced customer satisfaction (CS). Moreover, Prebensen and Xie (2017) tested the perceived value in experiential consumption. Subsequently, the perceived value was identified as an important driving force leading to consumer satisfaction. Hollebeek and Rather (2019) identified the direct effect of customer co-creation on CS.

More specifically, regarding each dimension of PCV, we expect a significant affection on CS. Customers tend to be more satisfied when they perceive more value from the service encounters (Sharma and Patterson, 1999; Patterson and Smith, 2001; Ouschan et al., 2006). The co-produced travel plan for an individual customer, due to the unique experience and customized characteristics, provides for a greater likelihood of transaction benefits, which increase the likelihood of CS.

Customer perceived values from an enjoyable and friendly interpersonal relationship further enhances CS in professional services (Sharma and Patterson, 1999; Patterson and Smith, 2001, 2003). In this study, customized tour arrangement requests high contact and pleasant interaction during the service encounter, which consequentially lead to customers’ sense of satisfaction.

Bruce (1998) defines satisfaction as “A state of mind that represents the composite of a user’s emotional and material responses to a particular activity, such as information seeking.” In addition, learning is positively linked to the implementation of customer information and knowledge increase. This study then argues that information seeking (e.g., travel consultation) can promote enjoyment and learning. That is, obtaining valuable reference information and knowledge acquisition influences CS with the activity. Thus, the following hypothesis are stated:

**H4.** Perceived co-creation of EV has a positive relationship with CS.

**H5.** Perceived co-creation of RV has a positive relationship with CS.

**H6.** Perceived co-creation of information/knowledge has a positive relationship with CS.

##### Indirect Relationship of Perceived Co-creation Values and Tourist Loyalty With Customer Satisfaction as a Mediator

There are fruitful studies that empirically test the relationship between satisfaction and loyalty. The significant role of CS in producing favorable outcomes is well documented in the marketing literature and appropriate to the present discussion. In marketing channels, Geyskens et al. (1999) conducted a meta-analysis of satisfaction, and satisfaction is found to be an antecedent to loyalty. Similar results appeared in retailing (Bloemer and Odekerken-Schroder, 2002), brand loyalty (Anjum et al., 2013), and hospitality settings (Singh and Sirdeshmukh, 2000). A closer preference fit of co-created products and services leads to positive customer attitudes, subsequent purchase intentions, willingness-to-pay, and referrals (Mathwick et al., 2007; Franke et al., 2009).

Additionally, scholars verified CS as a mediate role to CL in the retailing industry, when they intend to explore customer’s shopping values (Vijay et al., 2019), service quality (Slack and Singh, 2020). Similar evidence is also found in the hospitality industry for testing hotel perceived value (El-Adly, 2019). We, therefore, expect the indirect relationship of PCVs and tourist loyalty with CS as a mediator. We posit:

**H7.** Customer satisfaction mediates the relationship between PCV and CL.

#### Conceptual Framework

**FIGURE 2:**
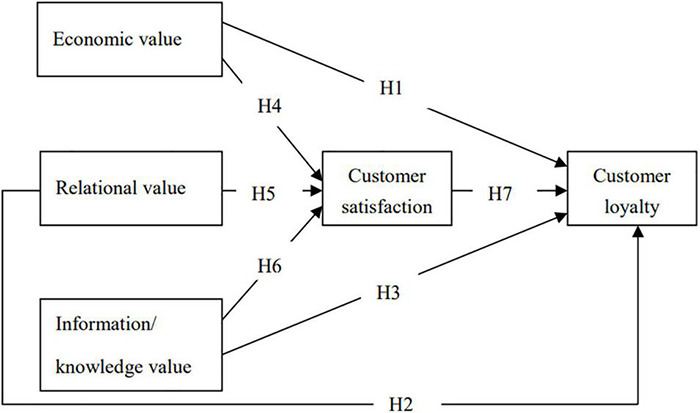
Conceptual framework.

Based on the above analysis, the SEM conceptual model of the relationship between PCVs and CL is shown in Figure 2.

#### Reliability and Validity of Model

The proposed conceptual framework has five observed variables: EV, RV, information/knowledge value, CS, and CL.

##### Exploratory Factor Analysis

Each construct was assessed from perspectives of factor loading, eigenvalue, and variance. The researcher measured the internal reliability for each factor identified in the exploratory factor analysis (EFA) process, calculating the Cronbach alpha to evaluate the reliability of a certain scale (Nunnally, 1994). Normally, a value of 0.7 for Cronbach’s alpha is satisfactory for scales. If a more contingent stringent measure was taken, item-to-total correlations could also be checked in assessing reliability. Usually, a value of item-to-total equal to 0.5 or above is deemed acceptable (Lankford and Howard, 1994). At the same time, Bartlett’s Test of Sphericity and KMO results are also reported in Table 4.

**TABLE 4:** EFA results.

Measurements	KMO	Chi-square	Eigen values	Variance explained	Coefficient α
EV	0.865	895.311	3.666	73.315	0.907
RV	0.731	348.632	2.307	76.894	0.849
IKV	0.890	903.508	3.727	74.545	0.913
CS	0.814	1172.542	3.730	74.603	0.913
CL	0.872	723.085	3.468	69.351	0.889
Bartlett’s Test of Sphericity: df = 10, Sig. = 0.000					
Rotation Method: Varimax with Kaiser Normalization. Rotation converged in 5 iterations					

*EV stands for economic values. RV stands for relational values. IKV stands for information/knowledge values. CS stands for customer satisfaction. CL stands for customer loyalty.*

EFA shows that the item-to-total correlations for all items were more than 0.7, indicating a reasonable fit. Both these factors have high Cronbach’s alpha above 0.8, indicating established internal consistency within the factor (Table 4).

##### Confirmatory Factor Analysis

To verify the underlying factor structure in the proposed scales from previous studies, the researcher performed Confirmatory Factor Analysis (CFA) in AMOS 23 to assess the reliability and validity of measurement scales. This study checked composite reliability, convergent validity, and discriminant validity.

The reliability of a scale refers to whether the scale is stable or consistent across varying situations. This study used Cronbach’s alpha coefficient, a popular diagnostic measure of reliability, to assess the internal consistency of the construct. With SEM models, a slightly different composite reliability is often used. Composite reliability is analogous to Cronbach’s alpha, with its calculation being based on both factor loadings and error variances of each item to a given factor. Its calculation formula was as follows, in which *Li* is the factor loading and *ei* is the error variance for each construct.


(1) $${\left(\sum_{i=1}^{n}L_{i}\right)}^{2}/{\left(\sum_{i=1}^{n}L_{i}\right)}^{2}+\left(\sum_{i=1}^{n}e_{i}\right)$$

A scale is reliable if the value of composite reliability equals or is greater than 0.6 (Bagozzi and Kimmel, 1995). During CFA, composite reliability and Cronbach’s alpha were calculated to assess the reliability of the construct. Table 5 presents the results and indicates the composite reliability for the five factors ranged from 0.812 to 0.902. For this study, Cronbach’s alpha values were all larger than 0.080. Therefore, good internal consistency among the variables was secured.

**TABLE 5:** Composite reliability.

Factors	Composite reliability	Cronbach’s alpha
EV	0.875	0.909
RV	0.812	0.851
IKV	0.867	0.914
CS	0.902	0.913
CL	0.860	0.893

*EV stands for economic values. RV stands for relational values. IKV stands for information/knowledge values. CS stands for customer satisfaction. CL stands for customer loyalty.*

Convergent validity evaluates the extent to which the common variance is shared among items of a specific construct (Hair et al., 2010). Several ways are available to measure the convergent validity. Fornell and Larcker (1981) recommended average variance extracted (AVE), which is a summary indicator of convergence. It is the average amount of variance in measurement items that a latent variable is able to explain and it can be calculated by:


(2) $${\left(\sum_{i=1}^{n}L_{i}\right)}^{2}/n$$

in which *Li* is the standardized factor loadings.

Besides AVE, Bagozzi and Yi (1988) suggested that factor loadings larger than 0.5 were predictive of acceptable convergent validity, which should be statistically significant. In this research, the AVEs of all constructs were above the critical value of 0.5, suggesting that these proposed items have captured more than 50% of the variances in the factors they intended to measure. The factor loadings of the items were greater than 0.5 and therefore were statistically significant (*p* < 0.001). These results indicated that the convergent validity of the constructs was satisfactory (see Table 6).

**TABLE 6:** Factor loading.

Measurements	Mean	S.D.	Factor loadings	AVE
EV	5.170	1.280	0.850	0.740
RV	5.180	1.240	0.870	0.770
IKV	4.850	1.330	0.860	0.750
CS	4.920	1.190	0.860	0.740
CL	4.980	1.220	0.830	0.700

***p < 0.001. EV stands for economic values. RV stands for relational values. IKV stands for information/knowledge values. CS stands for customer satisfaction. CL stands for customer loyalty.

Discriminant validity is the degree to which two measures are different from each other. In general, inter-correlations among variables lower than 0.85 indicate acceptable discriminant validity (Kline, 2005). Another more stringent criterion for adequate discriminant validity maintains that the squared root of AVE for each construct should exceed the correlation coefficients of the corresponding inter-constructs (Fornell and Larcker, 1981). The results of discriminant validity shown in Table 7 suggested satisfactory discriminant validity, as the square root of the AVE estimates is larger than any inter-correlation coefficients. This indicated that each variable is more highly correlated with its measurement items than any others. Furthermore, it was found in the current study that all inter-variable correlations were lower than 0.850. Therefore, the discriminant validity of the proposed scale was established.

**TABLE 7:** Inter-variable correlations.

	EV	RV	IKV	CS	CL
EV	**0.857**				
RV	0.843	**0.877**			
IKV	0.628	0.607	**0.865**		
CS	0.642	0.585	0.607	**0.863**	
CL	0.708	0.684	0.493	0.653	**0.837**

*Inter-correlation coefficients are below the diagonal and squared root of AVE estimates are presented on the diagonal. EV stands for economic values. RV stands for relational values. IKV stands for information/knowledge values. CS stands for customer satisfaction. CL stands for customer loyalty.*

*Bold values represent the inter-correlation coefficients of each variable.*

#### Structural Equation Model

**FIGURE 3:**
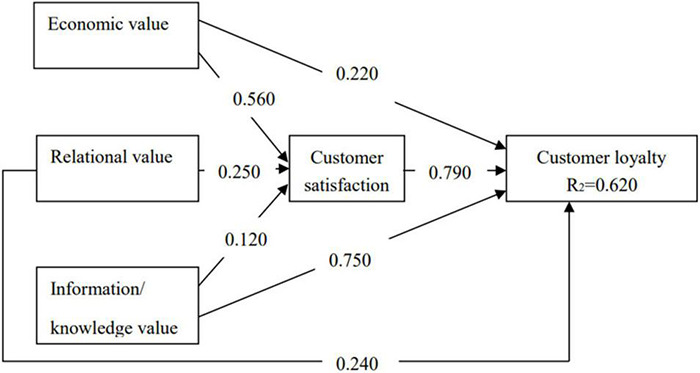
Structural model.

Structural models examine the causal relationships among latent variables (Byrne, 2010). This is where multiple regression analyses examine inter-relationships among latent variables and test proposed hypotheses. As proposed in the conceptual framework, Figure 3 reports the analysis results and the overall fit of the proposed data to the collected data.

Fit statistics:

χ^2^ = 2071.500, df = 465, *p* < 0.0001

Default root mean square residual (RMR) = 0.069

Root mean square error of approximation (RMSEA) = 0.069

Goodness-of-fit index (GFI) = 0.856, normed fit index (NFI) = 0.902, IFI = 0.992, Tucker-Lewis index (TLI) = 0.991, and comparative fit index (CFI) = 0.922

The researcher tested the proposed model using AMOS. The model fit indices [χ^2^ (df) = 2071.550 (465); RMR = 0.069; GFI = 0.856; NFI = 0.902; IFI = 0.992; TLI = 0.991; CFI = 0.922; and MSEA = 0.069] suggested that the proposed model examining the inter-relationships among the latent variables had an acceptable fit to the data.

#### Hypotheses Testing

Given an acceptable goodness-of-fit of the structural model, the researchers examined the proposed hypotheses among the latent constructs.

Hypotheses 1–3 posited that PCVs [EVs, RVs, and information/knowledge values (IKVs)] would have a positive and direct influence on tourist’s loyalty. The AMOS outputs suggested that these relationships were statistically significant (*p* < 0.001). The standardized coefficients were 0.220, 0.240, and 0.750, respectively, which signals positive influences of PCVs on CL as predicted in the hypothesis. As such, Hypotheses 1–3 were supported.

Hypotheses 4–6 proposed that PCVs enhance CS. The standardized coefficient of 0.560, 0.250, and 0.120 implied that all PCVs positively influence customers’ feeling of satisfaction.

Hypothesis 7 proposed that customer’s satisfaction would mediate the relationship between each PCV and CL. The standard coefficient was 0.790 indicating that customer’s satisfaction has a strong impact on their loyalty. Therefore, Hypothesis 7 was supported.

In summary, with regard to the hypotheses as set out in Figure 3, the standardized path coefficients and *t*-values of all of the relationships hypothesized in the model are presented in Table 8. The standardized coefficient showed the resulting change in an endogenous variable from a unit change in an exogenous variable, with all other exogenous variables held constant. The *t*-value indicates whether the corresponding path coefficient is significantly different from zero. Coefficients with *t*-values ranging between –1.96 and +1.96 are statistically insignificant. This implied that there is a high chance of obtaining a relationship of this magnitude purely by sampling error. In the present study, the coefficients of *t*-values were ranging from 5.390 to 20.663, which indicate all proposed hypotheses were statistically supported.

**TABLE 8:** Test of hypotheses.

Hypothesis	Path	Standardized coefficient	*t*-value	Result
H1	EV → CL	0.220	5.390	Supported
H2	RVp CL	0.240	5.315	Supported
H3	IKVp CL	0.750	10.916	Supported
H4	EV → CS	0.560	8.207	Supported
H5	RV → CS	0.250	3.496	Supported
H6	IKVp CS	0.120	3.483	Supported
H7	CS → CL	0.790	20.663	Supported

*EV stands for economic values. RV stands for relational values. IKV stands for information/knowledge values. CS stands for customer satisfaction. CL stands for customer loyalty.*

#### Direct and Indirect Effects of Perceived Co-creation Value on Customer Loyalty

In general, the model, as shown in Figure 3, has a squared multiple correlation (*R*^2^) of 0.62 indicating that PCV predicts 62% of the variance in tourist loyalty. The direct effect of EV on CL is 0.220, the direct effect of RV on CL is 0.240, and the direct effect of IKV on CL is 0.750. In terms of the indirect effect, the path coefficient between EV and CS is 0.560, between RV and CS is 0.250, and between IKV and CS is 0.120. In addition, the path coefficient between CS and CL is 0.790. This explains the mediating model of PCV on CL. The results revealed that customer perceives values during the service procedure positively influences their loyalty toward the service supplier through their satisfaction.

## Conclusion

This study, motivated by previous research on customer-service provider co-creation activity (Grissemann and Stokburger-Sauer, 2012), empirically develops a measurement scale of PCV and investigates a model of value co-creation formation mechanism in tourism services.

It is sought to identify the underlying dimension of PCVs in the tourism industry. In relationship marketing, co-creation values as an important construct have generally been believed as an essential factor that determines service success (Vargo and Lusch, 2004; Yi and Gong, 2013). The growing interest in the importance of co-creation values in professional service setting requires a closer examination of its conceptual and statistical dimensions (Chan et al., 2010). To contribute to the existing body of knowledge concerning relationship marketing, this study developed and validated a PCV scale in the service context of the tourism industry service context. The result is consistent with the previous research findings in other fields. Collectively, the pre-existing two dimensions of EV and RV together with the underlying dimension of information/knowledge value represent the fundamental building blocks of co-creation values in the customized tour arrangement service setting.

The presented work examined the causal relationships of latent variables composed by PCVs, CS, and CL, respectively. The propositions of hypothesis are statistically supported, moreover, CS is identified as an important mediate impact factor between the two latent variables of PCVs and CL. It generates a better understanding of customer psychology and behavior during the high contact interaction between service provider and customer. Besides, the overall goodness-of-fit of the proposed model is statistically tested.

The study generates important implications both theoretically and practically. The findings cannot only benefit tourism scholars to extend the academic understanding in this line of research, but also help hospitality practitioners to better understand co-creation activity and its positive outcomes, which determine the possibility to retain and cultivate loyal customers and thus gain competitive advantage.

## Theoretical Implications and Managerial Implications

### Theoretical Implications

Primarily, within the context of customized tour arrangement service, this study, particularly from customer perspective, encourages customer to evaluate the whole service process. Chan et al. (2010) regarded company support while Grissemann and Stokburger-Sauer (2012) considered customer participation as the premise of co-creation value. This study involved the support of service provider and customer participation, a dynamic process-based framework is proposed to explore co-creation values and its mechanism chain. Critically important variables are integrated into a single model, which could advance the understanding of these constructs and their linkages. Thus the new contributions to this field of research.

Subsequently, research from Chan et al. (2010) confirmed EV and RV for both customer and service supplier as the valuable outcome of co-creation activity in the professional finance service context. This study specifically identified the underlying dimension of PCV in the context of China’s customized tour arrangement services. The associated validity and reliability were statistically established. The new dimension-IKV, together with the pre-existing two dimensions of EV and RV, represented the construct of PCVs in the customized tour service context, which extends academic understanding in this line of research.

Finally, the study contributes to research studies on the resource exchange theory, social exchange theory, and social identity theory in a value co-creation of China’s tourism context. (1) Resource exchange theory: By applying Foa and Foa (1974) resource exchange theory into value co-creation to examine the types and patterns of exchanges involved in interpersonal interactions. Knowledge and collaboration information as a valuable resource are shared and exchanged during service process is for a better travel plan development. Moreover, in this process, besides knowledge, experience, and information, the exchanged resources are made in terms of a monetary transaction of product and service. (2) Social exchange theory: Hare et al. (1959) social exchange theory is a highly regarded theory to explain why customers are willing to be involved in exchange relationships. It is also the theory that links co-creation activity to valuable outcomes in this study. Social exchange theory helps to understand the importance of customer-company interactions in the light of value co-creation (Grissemann and Stokburger-Sauer, 2012). Particularly in this study, value co-creation activities launched by service provider can be regarded as exchange initiatives or activities, support from the tour company is regarded as input into the social exchange process that drives customers to believe in their exchange partner, thereby causing them to reciprocate with positive behavioral outcomes. (3) Social identity theory: According to social identity theory (Tajfel and Turner, 1979), people’s wellbeing and behaviors are implicated by their knowledge and emotion attached to their group memberships. Various social benefits are generated to customers during co-creation procedures, which help to enhance customers’ social status, as other stakeholders would regard them as a valuable information source. Besides, customer’s communication skills, social contacts, and enjoyment can be enhanced by their active participation in interpersonal activities (Etgar, 2008). In line with Grissemann and Stokburger-Sauer (2012), when customers engage in their premium travel package development process, they may feel pride and belong to the company owing to the co-created accomplishment. A self-designed travel plan can be viewed as a successful outcome that makes customer enjoyable, this sense of self-achievement can further influence a series of customer perceptions and behaviors, such as satisfaction and repurchase intention.

### Practical Implications

Several crucial implications can be derived from the study model and findings for marketing and tourism practitioners. First, in co-creation activity, company support and customer participation are equally important. On the one hand, company support represents by emphasizing the employees performing all their skills, even extra efforts, to meet the needs of the customer when the firms want to deliver values to their customer (Homburg et al., 2009a,b). Evidence shows that premium service capabilities to fit customers’ individual preferences contribute to a higher potential of competitive advantage (Zhang and Chen, 2008). On the other hand, the benefits of customer engagement are perfectly embodied when applied in co-creation activity. In this case, the more the customers engaged in their travel package co-producing, the more values they perceive, and consequently reflect their satisfaction and loyalty. Thus, firms can take these findings as references when considering customer empowerment strategies (Füller et al., 2009; Grissemann and Stokburger-Sauer, 2012).

Moreover, the study emphasizes the better understanding of firm-customer communications, this is in line with Lusch and Vargo (2006)’s service-dominant (S-D) logic, which proposes that value is not only created by the provision of the service itself, but during the actual service development process. In this sense, the co-created value requires an active dialog which should not just be the standardized operation, but rather be the customer tailored communication. In the travel agency services, the study found that high-quality communication can be reflected in cooperating with each customer to find the most personalized transportation, accommodation, and entertainment program.

Finally, the professional service settings are usually more complex, which customers are less familiar with (Bitner et al., 1994). Thus, to ensure positive co-creation outcomes, customers also need to be trained to know how to behave and what to expect in given situations. Employees are required to devote more efforts to help the customer visualize the values. EV, as a direct benefit, not only strengthens the motivation of customer engagement, but also generates a competitive advantage. Relationship value alone may not link customers permanently to the company, but it is hard for competitors to imitate. Moreover, the experiences of both sides of customers and employees can be enriched in terms of information and knowledge exchanging during the service process. Therefore, employees must realize the business value of the new approach and their responsibilities to coordinate with customers in co-creation service encounters. The solid instrument of PCV might contribute to help the firm for a better service platform designing and the strategies for the firms’ consideration, altering policies for employee recruiting, training, and rewarding.

## Limitations and Future Research Directions

By applying co-creation in a tourism service setting, although this study generated considerable insights, there still exist some limitations that should be acknowledged. First, the generalizability of the study findings should also be considered as one of the study’s limitations. Although this study applied careful selection of a sampling frame from reliable sources, the research model was tested in one customized tour product with two travel companies only. Thus, applying the recommendations of Grissemann and Stokburger-Sauer (2012), testing the model in differentiated tourism service settings, the results might be affected. Future research could be extended to other sectors under the hospitality tourism umbrella (e.g., hotels, restaurants, retail businesses, cruise lines, and other tour operators) to draw a holistic picture for the tourism industry.

Second, the dimensionality of each construct needs to be continuously developed for further conceptualization. “Only by narrowing down their area of research, and thus a new specialty, are scientist able to effectively manage the continuously growing literature” (Wray, 2005, p. 153). This study does not consider impacts from other specific potential factors and the possible inter-relationships among these influencing factors. For example, the degree of company support (Hoyer et al., 2010) and the level of customer involvement (Sharma and Patterson, 1999) are two premises of co-creation that deserve more observation. Further efforts could be made in this direction and other potential motivational factors.

Additionally, regarding the relationship-building component, customer and employee’s attitudes, and emotional responses are likely to affect each other in the dynamic co-creation activity. Whether co-created values exist in other terms, it is believed that longitudinal studies would help to have it clarified. Thus, future studies could elaborate on the variables of PCVs to develop liable and valid multi-item scales or go to the second-order of the latent variable.

## Data Availability Statement

The original contributions presented in the study are included in the article/supplementary material, further inquiries can be directed to the corresponding author/s.

## Author Contributions

KT contributed to the idea development, conceptualization, literature review, survey development, qualitative data collection and analysis, writing-up original draft, review and editing, and revision of the study. JY contributed to the quantitative research framework conceptualization, survey development, quantitative data collection and analysis, and revision of the study. PC contributed to the overall research guidance, idea development, conceptualization, survey development, and revision of the study. HX contributed to the database organization, statistical analysis, and revision of the study. All authors contributed to the article and approved the submitted version.

## Conflict of Interest

The authors declare that the research was conducted in the absence of any commercial or financial relationships that could be construed as a potential conflict of interest.

## Publisher’s Note

All claims expressed in this article are solely those of the authors and do not necessarily represent those of their affiliated organizations, or those of the publisher, the editors and the reviewers. Any product that may be evaluated in this article, or claim that may be made by its manufacturer, is not guaranteed or endorsed by the publisher.
